# The complete mitochondrial genome of the neotropical helicopter damselfly *Megaloprepus caerulatus* (Odonata: Zygoptera) assembled from next generation sequencing data

**DOI:** 10.1080/23802359.2016.1192504

**Published:** 2016-07-23

**Authors:** Wiebke Feindt, Hans-Jürgen Osigus, Rebecca Herzog, Christopher E. Mason, Heike Hadrys

**Affiliations:** a ITZ, Division of Ecology and Evolution, University of Veterinary Medicine Hannover, Hannover, Germany;; b The Department of Physiology and Biophysics, The HRH Prince Alwaleed Bin Talal Bin Abdulaziz Alsaud Institute for Computational Biomedicine, Weill Cornell Medical College, New York, NY, USA;; c The Feil Family Brain and Mind Research Institute, Weill Cornell Medical College, New York, NY, USA;; d Sackler Institute for Comparative Genomics, American Museum of Natural History, New York, NY, USA

**Keywords:** Mitochondrial genome, Odonata, *Megaloprepus caerulatus*, Zygoptera, s4 intergenic spacer

## Abstract

Odonata (dragonflies and damselflies) is a small order at the base of flying insects (Pterygota). Resolving family-level phylogenetic relationships within this order receives great attention. Hereby, genetic data already resulted in various changes, which are however still under discussion. Mitochondrial genomes may further enhance such phylogenies. This study presents the complete mitochondrial genome of the Neotropical damselfly *Megaloprepus caerulatus* based on next generation sequencing (NGS) data on total genomic DNA. The total length comprises 16,094 bp and includes the standard metazoan set of 37 genes together with a 1376 bp long A + T rich (control) region. Gene content, gene arrangement and base frequency are consistent with other odonate mitochondrial genomes. It further contains four intergenic spacer regions, indicating a possible family specific feature for the Coenagrionidae and its close relatives.

The relatively small insect order Odonata owns a key position in the evolution of winged insects and as sensitive indicator organisms for freshwater ecosystems. Robust phylogenies are one ultimate requirement for studies in evolution, ecology and developmental biology. Over the last 10 years many molecular data-based attempts have already resulted in various reorganizations of phylogenetic relationships (e.g. Dumont et al. 2010; Carle et al. 2015). However, today’s phylogenies still harbour open questions concerning family-level as well as deeper taxonomic positions. Mitochondrial genome projects in odonates may help to unravel such unresolved phylogenetic relationships by constructing more robust phylogenies based on complete gene and genome comparison (e.g. Simon & Hadrys 2013). One family that has received great attention in the past are the Pseudostigmatidae (giant damselflies) (e.g. Groeneveld et al. 2007; Ingley et al. 2012). They were recently placed into the Coenagrionidae based on three sequence markers (Dijkstra et al. 2014). Here, more robust genomic data are needed not only to clarify their taxonomic position but also to facilitate genomic-based studies on Neotropical forest health indicators. In this work, we present a complete mitochondrial genome of *Megaloprepus caerulatus* as the first member of this group*. Megaloprepus caerulatus* was already included in many ecological (e.g. Fincke & Hedström 2008) and evolutionary studies in tropical habitats, but yet little research has been done on its genetics (e.g. Fincke & Hadrys 2001; Feindt et al. 2014).

Via the standard phenol–chloroform extraction, (Hadrys et al. 1992) total genomic DNA from the flight muscles of a single *M. caerulatus* individual collected at the Biological Research Station La Selva (OTS), Costa Rica (N 10°25′19.74″W 84°00′35.22″) was extracted. Library preparation and DNA sequencing (100 bp mate pairs with different insert sizes, Illumina HiSeq2500, Illumina Inc., San Diego, CA) was performed at the Weill Cornell Medical College in New York. The mitochondrial genome was assembled using Geneious vers. 8.1 (http://www.geneious.com); while mapping a fraction of the cleaned reads onto a seed sequence (here, *cox1*: KF895301.1 and *nad1*: KF895193.1) allowing strand extension using varying iterates. Hereby, the settings included a minimum overlap of 60 bp, a minimal overlap identity of 90%, and a variable word size between 35 and 50. The mitochondrial genome was annotated using the MITOS WebServer (mitos.bioinf.uni-leipzig.de/index.py) and verified via BLAST (Altschul et al. 1990) against the NCBI database (http://www.ncbi.nih.gov) or additionally with published mitochondrial genomes of other odonate species. Transfer RNA genes were identified by a tRNA covariance model implied on the tRNAscan-SE vers. 1.21 Search Server (http://lowelab.ucsc.edu/tRNAscan-SE; Lowe & Eddy 1997) and ARWEN vers. 1.2 (http://mbio-serv2.mbioekol.lu.se/ARWEN, Laslett & Canbäck 2008). Phylogenetic relationships were reconstructed using a selection of odonate mitochondrial genomes and a mayfly *Parafronurus youi* as outgroup (Figure 1). All 13 protein coding genes and rRNA genes were aligned independently, then concatenated and a maximum parsimony tree was calculated (1000 replicates) in PAUP (Swofford 2002).

**Figure 1.**
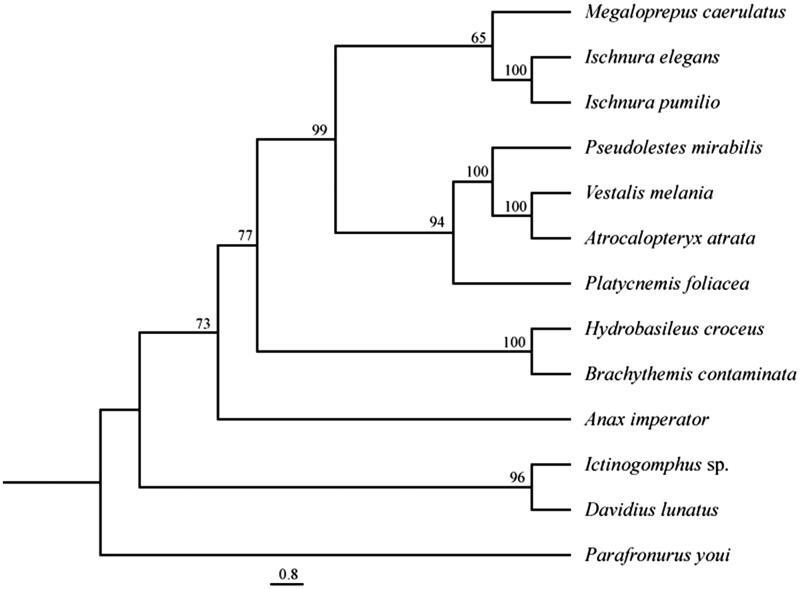
Phylogenetic relationships of odonate species based on maximum parsimony analysis of concatenated mitochondrial protein coding genes and rRNA sequences: *Brachythemis contaminata* (NC_026305), *Ictinogomphus* sp. (KM244673), *Hydrobasileus croceus* (NC_025758), *Davidius lunatus* (NC_012644), *Ischnura pumilio* (NC_021617), *Pseudolestes mirabilis* (NC_020636), *Atrocalopteryx atrata* (NC_027181), *Vestalis melania* (NC_023233), *Platycnemis foliacea* (NC_027180), *Anax imperator* (KX161841), *Ischnura elegans* (KU958378), and *Parafronurus youi* (EU349015.1) as outgroup. The heuristic search (under the 50% majority-rule with 1000 bootstrap replicates) placed *Megaloprepus* as a sister species to *Ischnura* spp, whereas relationships are displayed as in other phylogenies.

The obtained complete mitochondrial genome of *M. caerulatus* (NCBI: KU958377) is the first of a tropical odonate species. It has a total length of 16,094 bp, and is the second largest aside of *Vestalis melania* (16,685 bp, NC_023233). In all observed parameters, the presented mitochondrial genome shows a strong similarity to all other already published odonate mitochondrial genomes (e.g. Lorenzo-Carballa et al. 2014; Tang et al. 2014; Yu et al. 2014; Chen et al. 2015; Feindt et al. 2016; Herzog et al. 2016). It contains the common arrangement of 37 genes including 13 protein-coding genes, 2 rRNA (16S and 12S rRNA) genes, 22 tRNA genes, and an A + T rich (control) region of 1376 bp in length. Comparing with the previously released single genes, we observed 100% similarity to *nad1* (DQ642992.1) (Groeneveld et al. 2007) and *16S* rRNA (DQ642987.1), and in *nad1* (JQ966612.1), *16S* (JQ966660.1) and *12S* rRNA (JQ966647.1) a similarity between 98% and 100% to genes illustrated in Ingley et al. (2012). The overall A + T content of the mitochondrial genome is 75.9% (A: 43.1%, C: 14.2%, G: 9.8%, T: 32.8%) and therefore it is similar to the base frequencies of the protein-coding genes (AT: 74.4%). Hereby, *atp8* encompasses the highest A + T content with 81.1% and *cox1*, as it was described in other odonates (Lorenzo-Carballa et al. 2014), with 68.9% the lowest. All protein coding genes start with characteristic invertebrate specific mitochondrial start codons: *cox1*, *atp6*, *cox3*, *nad4*, *nad4L* and *cob* use ATG; *nad2*, *cox2* and *nad6* start with ATA; *nad3* and *nad1* start with TTG; *nad5* starts with ATT and *atp8* starts with ATC. The standard stop codon TAA was used eight times (*nad2*, *cox1*, *atp8*, *atp6*, *nad4L*, *nad6*, *cob*, *nad1*), whereas TAG was used only once by *nad3*. An incomplete stop codon with a single T was found in four cases (*cox2*, *cox3*, *nad5* and *nad4*). Length of the tRNA genes in *M. caerulatus* ranges from 64 bp to 74 bp except for *trnL1* (Leu), *trnS1* (Ser), *trnG* (Ser) all of which employ the typical clover-leaf secondary structures.

A difference in numbers of intergenic spacer regions is described in the two large odonate orders (Lorenzo-Carballa et al. 2014). They seem to provide a phylogenetic signal for the split of Anisoptera (dragonflies) from Zygoptera (damselflies). The lack of the intergenic spacer region *s5* seems to be a damselfly specific character. In *M. caerulatus*, we detected four spacer regions at *trnY*/*cox1*, *trnF*/*nad5*, *trnT*/*trnP* and *trnS2*/*nad1* with a total length of 106 bp. With this we proved that the unique spacer *s4* between *trnF* and *nad5*, which is also present in *I. pumilio* (NC_021617), *Pseudolestes mirabilis* (NC_020636) and *I. elegans* (KU958378) might not only be a specific character for the Coenagrionidae. The phylogenetic tree (Figure 1) places *M. caerulatus* as a sister species to *Ischnura* spp. with low support. More mitochondrial genomic data is needed to resolve relationships within and between families. However, the mitochondrial genome presented in this study is a valuable resource for future population genomic studies and also owns potential for answering phylogenetic questions on higher taxonomic levels.
